# Dynamics of male meiotic recombination frequency during plant development using Fluorescent Tagged Lines in *Arabidopsis thaliana*

**DOI:** 10.1038/srep42535

**Published:** 2017-02-13

**Authors:** Fan Li, Nico De Storme, Danny Geelen

**Affiliations:** 1 *In vitro* Biology and Horticulture, Department of Plant Production, Faculty of Bioscience Engineering, Ghent University, Ghent, 9000, Belgium

## Abstract

Meiotic homologous recombination plays a central role in creating genetic variability, making it an essential biological process relevant to evolution and crop breeding. In this study, we used pollen-specific fluorescent tagged lines (FTLs) to measure male meiotic recombination frequency during the development of *Arabidopsis thaliana*. Interestingly, a subset of pollen grains consistently shows loss of fluorescence expression in tested lines. Using nine independent FTL intervals, the spatio-temporal dynamics of male recombination frequency was assessed during plant development, considering both shoot type and plant age as independent parameters. In most genomic intervals assayed, male meiotic recombination frequency is highly consistent during plant development, showing no significant change between different shoot types and during plant aging. However, in some genomic regions, such as I1a and I5a, a small but significant effect of either developmental position or plant age were observed, indicating that the meiotic CO frequency in those intervals varies during plant development. Furthermore, from an overall view of all nine genomic intervals assayed, both primary and tertiary shoots show a similar dynamics of increasing recombination frequency during development, while secondary and lateral shoots remain highly stable. Our results provide new insights in the dynamics of male meiotic recombination frequency during plant development.

During plant sexual reproduction, parental genomes reshuffle through a specialized type of cell division, e.g. meiosis, which reduces the somatic chromosome number to yield haploid cells and produces gametes with varying combinations of parental genetic information. A key event in the first meiotic cell division is the formation of crossovers (COs) between homologous parental chromosomes, which is tightly regulated by a series of consecutive events; including homolog recognition, pairing, synapsis, and recombination^1^. Meiotic CO formation is not only necessary for creating new allele combinations and increasing genetic diversity, but also is essential to ensure accurate chromosome segregation during meiosis I. Each CO acts as a tether between a pair of homologs, ensuring proper alignment and balanced segregation of resulting bivalents at metaphase I and anaphase I, respectively, by regulating correct attachment of corresponding kinetochores to the MI spindle^2^,^3^,^4^,^5^. Due to its central role in creating genetic variability, meiotic homologous recombination is considered crucial for plant evolution and speciation, and hence also constitutes an essential biological process for the development of novel crop varieties.

Experimentally, several methods exist for assessing patterns and rates of meiotic recombination. In general, they can be classified into two major categories: (1) cytological analysis of meiocytes using microscopy and (2) population-wide segregation analysis of genetic markers^6^,^7^,^8^,^9^. As an alternative method, Francis *et al*.^10^ developed a series of pollen-specific fluorescent markers (FTLs; fluorescent tagged lines) in the model plant *Arabidopsis thaliana*, that upon combination in a hemizygous state, allow for high-throughput quantification of male meiotic recombination in corresponding genomic intervals^11^,^12^. The FTLs contain linked transgenes (T-DNA inserts) that encode either DsRed, eYFP and eCFP in mature pollen grains through activation by the post-meiotic pollen-specific *LAT52* promoter^10^,^12^,^13^. Moreover, since FTLs are designed to contain two or three different linked fluorescent markers in a hemizygous state, the segregation pattern of fluorescent markers in resulting pollen grains also allows for the detection and quantification of CO interference and events of gene conversion^11^. Pollen-based analysis of male meiotic recombination using FTLs is generally performed using one of two following methods; namely (1) tetrad-based analysis using epi-fluorescence microscopy^10^,^11^,^14^ and (2) single-pollen analysis using flow cytometry^12^. For tetrad-based analysis, the methodology takes the advantage of the *Arabidopsis quartet1-2 (qrt1-2*) mutant (Figure S1), in which the four pollen grains remain physically attached to each other (tetrad) throughout the whole process of male gametogenesis due to lack of the pectin methylesterase enzyme^15^. Alternatively, FTL-based quantification of meiotic recombination frequency can also be performed based on the fluorescent analysis of single pollen grains by flow cytometry (Figure S1). Using this methodology, data acquisition speed can be increased up to 150-fold compared to tetrad analysis, allowing rapid quantification of male meiotic CO rates^12^. Both methods have their own specific advantages and disadvantages for meiotic recombination analysis. Tetrad-based analysis enables the detection of unviable or ectopically non-fluorescent pollen grains and thus allows for the elimination of related biases in meiotic CO analysis. At the downside, this method is highly laborious and time-consuming, and may suffer from detection errors due to manual counting or interpretation. In contrast, single pollen grain analysis using flow cytometry allows high-throughput quantification of meiotic CO rates and excludes the possibility of human errors. Moreover, this method generates far more data points, improving statistical reliability. As a major drawback, flow cytometry-based FTL analysis does not allow for detection of alterations in pollen viability and related changes in fluorescence intensity, putatively leading to non-registered biases in recombination frequency measurement. Currently, there are 113 single FTL markers available, including 35 DsRed, 41 eYFP and 37 eCFP fluorescent markers, which are randomly spread across the five *Arabidopsis* chromosomes^11^. By combining these fluorescent markers in double or triple ‘linked’ configurations, this extensive set of FTLs provides a flexible way to assay the male meiotic recombination rate in different intervals of the *Arabidopsis* genome.

In *Arabidopsis*, meiotic recombination frequency varies during plant development^16^. Pollen tetrad analysis in the genomic intervals I1a and I3a revealed that crossover frequency in the second and third branches are significantly higher than that of the main shoot, which indicates that developmental position can affect crossover frequencies during male gametogenesis^10^. In addition, a whole-genome level study of meiotic CO frequency in *Arabidopsis thaliana* revealed that aging affects CO frequency during megaspore formation (increased 29.86%), but not during pollen formation^17^. By analyzing the data of main and lateral shoots, it was indicated that this age effect correlates to the developmental age of each shoot rather than that of the whole plant^17^. In *Arabidopsis*, flowers develop on different shoot types, such as primary shoot, secondary shoot, tertiary shoot and lateral shoot (Fig. 1), in which meiotic recombination occurs independently. Up till now, only for interval I1a and I3a, the developmental position was shown to have a significant effect on the meiotic CO frequency^10^. However, in general, there is no clear insight into whether there exists variability in the male meiotic CO frequency during plant aging and shoot development. In this study, we use nine independent FTLs intervals (I1a, I1b, I2a, I2b, I3c, CEN3, I5a, I5c and I5d) to measure the spatio-temporal dynamics of male CO frequency in flowers isolated from primary, secondary, tertiary and lateral shoots, in order to provide an extensive overview of the spatio-temporal dynamics of male meiotic recombination frequency in the genomic intervals delineated by a set of widely used pollen FTL markers.

## Results

### Fluorescent Tagged Lines show partial loss of pollen-specific fluorescence

FTL-based quantification of meiotic recombination frequency in *Arabidopsis* relies on the segregation analysis of two or three linked hemizygous fluorescent markers (DsRed, eYFP and eCFP) in mature pollen grains. Due to their transgenic nature, these recombinant transgene encoding proteins may be prone to epigenetic silencing, reducing the accuracy of associated meiotic CO read-out. In order to assess putative losses or biases in pollen fluorescence of the widely used FTL marker combinations (including I1a, I1b, I2a, I2b, I3c, CEN3, I5a, I5c and I5d), we monitored DsRed, eYFP and eCFP expression in tetrad configured pollen grains isolated from *quartet1-2*^*−/−*^ (*qrt1-2*^*−/−*^) plants containing homozygous FTL marker combinations.

Theoretically, in the homozygous FTL tetrad-pollen, all four pollen grains enclosed should express the corresponding fluorescent markers. However, in all lines analyzed, we observed a combination of fluorescent and non-fluorescent pollen grains, suggesting that some pollen grains were either dead or suffered from a suppressed expression of the reporter construct. In our study, pollen grains are classified ‘dead’ when all fluorescent markers enclosed fail to express, irrespective whether the pollen is shriveled up or fully developed. In contrary, pollen showing specific loss of one or two fluorescent reporter(s), whereas the remaining other(s) are still expressed, are classified as ‘loss of fluorescence’. Based on these definitions, we quantified the marker-specific loss of pollen fluorescence and found that all FTL lines examined show a certain level of fluorescence ‘silencing’ in corresponding DsRed, eYFP and eCFP markers (Fig. 2, Table 1 and S1). Although the level of fluorescence ‘silencing’ in most FTL markers are generally low (ranging from 0.09% to 3.77%), some FTL markers show a more extreme loss of fluorescence. For example, the I1a line shows an extremely high level of fluorescent loss in DsRed marker (19.0% ± 3.68%).

### Male meiotic CO rate dynamics varies between genomic regions during plant development

We measured male meiotic recombination frequency at nine different genomic intervals in different shoot types at different time points during *Arabidopsis* plant development (Figure S2 and Table S2). Since the nine intervals comprise different sized genomic regions, we used ‘CO rate per standard unit DNA sequence’ (cM/Mb) to calculate the recombination frequency respective to the genomic size. To examine whether plant age, independent of shoot type, influences male meiotic recombination frequency, we compared the average recombination frequency of all shoots present at the same time during four subsequent weeks (Fig. 3). The results show that the genetic distance in four of the nine genomic intervals significantly varies according plant age [I1a (ANOVA: F = 6.5510, *p* = 0.0151), I1b (ANOVA: F = 19.8920, *p* = 0.0005), I3c (ANOVA: F = 28.1260, *p* = 0.0001) and I5a (ANOVA: F = 70.7510, *p* = 0.0001), *p* value was calculated with post-hoc Tukey HSD test (α = 0.05)]. There show a gradual increase of recombination rate during flowering in I1b and I3c interval, but is not that clear in the I1a and I5a. The other five intervals do not display any significant variation in male meiotic recombination frequency during plant aging. This indicates that plant age selectively affects male meiotic recombination frequency only in specific genomic regions, and that this age effect is rather minor.

### Male meiotic CO rate in most genomic regions are not influenced by shoot type

To assess the effect of shoot type on the male meiotic recombination rate in different genomic regions, the mean CO rate during the entire development of each shoot type was assessed in nine different genomic intervals (Fig. 4). Comparative analysis revealed that three intervals exhibit a significant difference in CO rate between the four shoot types [I1a (ANOVA: F = 12.8200, *p* = 0.0020), I2a (ANOVA: F = 5.5220, *p* = 0.0238) and I5a (ANOVA: F = 17.6830, *p* = 0.0007), *p* value was calculated with post-hoc Tukey HSD test (α = 0.05)]. In the genomic interval I1a, for example, the male meiotic recombination frequency in the tertiary shoot is significantly higher than that of other shoot types. These results indicate that the developmental position (shoot type) affects male meiotic recombination frequency in specific genomic regions during *Arabidopsis* male sporogenesis. However, this is not common to all genomic regions since the other six intervals tested do not show any significant difference in the male meiotic CO rate between the different shoot types.

### Male meiotic CO rate increase in primary and tertiary shoots during plant development

**Figure 1:**
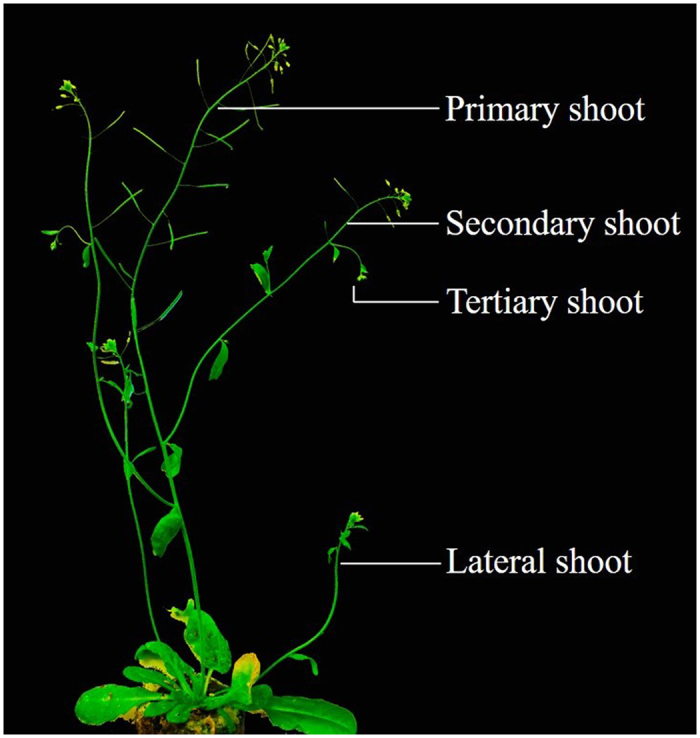
Arabidopsis model showing different shoot types. Primary shoot is the main shoot of plant branching into secondary shoots which in turn branch into tertiary shoots. The lateral shoot develops from the axils of rosette leaves.

**Figure 2:**
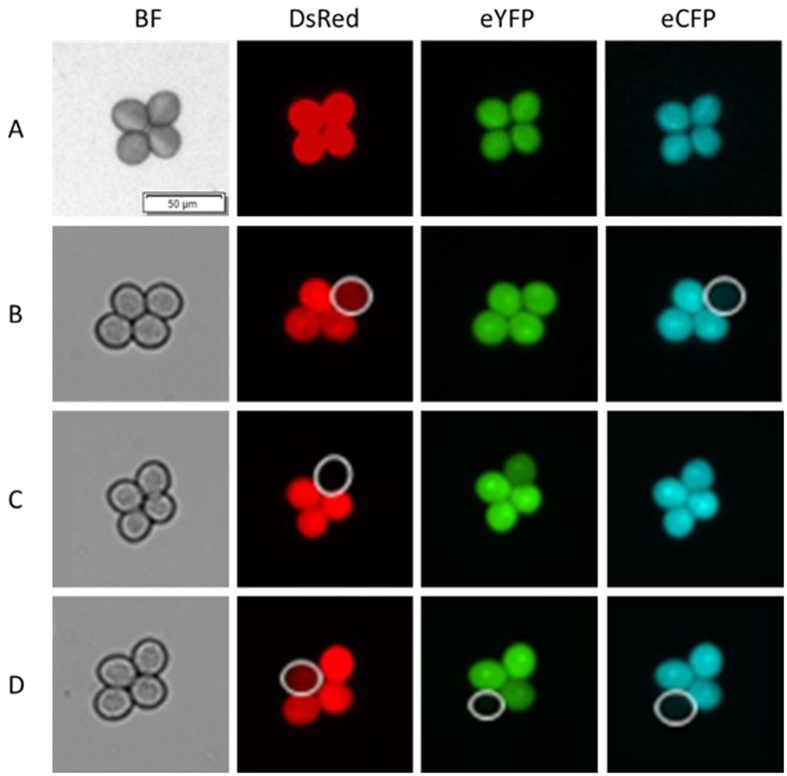
Expression analysis of pollen-specific fluorescence in the homozygous FTL line I2ab containing both DsRed, eYFP and eCFP in the *qrt1-2*^*−/−*^ background show variable loss of fluorescence. (**A**) A tetrad-pollen show full expression of DsRed, eYFP, and eCFP fluorescence; (**B**) A tetrad-pollen show fluorescent loss of eCFP and weak DsRed expression; (**C**) A tetrad-pollen exhibit fluorescent loss of DsRed and weak eYFP expression; (**D**) A tetrad-pollen show weak DsRed expression in one pollen grain, and fluorescent loss of eYFP and eCFP in another pollen grain. The white circles indicate the pollen grains which lost fluorescence. Scale bar also indicated in 50 μm.

**Figure 3:**
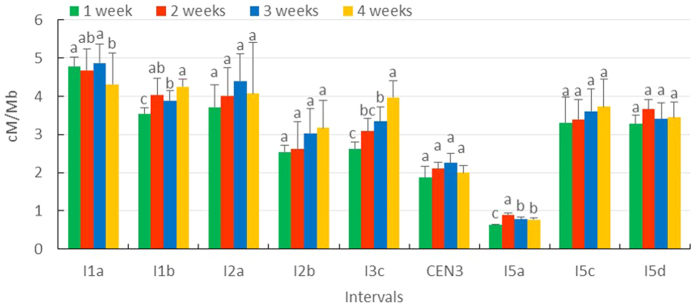
Mean recombination frequency of all shoots present at the same time during four subsequent weeks following flowering induction in different genomic intervals. The ‘number of weeks, in the legend refers to the number of week after flowering induction. Statistical significance was analyzed based on one-way ANOVA [I1a (F = 6.5510, *p* = 0.0151), I1b (F = 19.8920, *p* = 0.0005), I2a (F = 1.3280, *p* = 0.3314), I2b (F = 1.8500, *p* = 0.2164), I3c (F = 28.1260, *p* = 0.0001), CEN3 (F = 2.5310, *p* = 0.1306), I5a (F = 70.7510, *p* = 0.0001), I5c (F = 0.6430, *p* = 0.6088) and I5d (F = 1.7630, *p* = 0.2317), *p* value was calculated with post-hoc Tukey HSD test (α = 0.05)].

**Figure 4:**
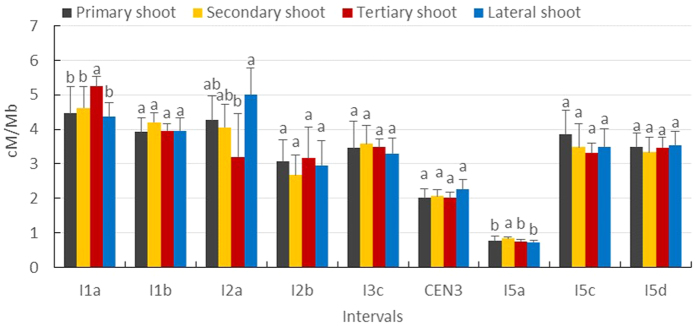
Mean recombination frequency during the entire development of each shoot type in nine different genomic intervals. Statistical significance was analyzed based on one-way ANOVA [I1a (F = 12.8200, *p* = 0.0020), I1b (F = 1.9040, *p* = 0.2074), I2a (F = 5.5220, *p* = 0.0238), I2b (F = 0.4580, *p* = 0.7193), I3c (F = 1.2450, *p* = 0.3561), CEN3 (F = 1.3560, *p* = 0.3236), I5a (F = 17.6830, *p* = 0.0007), I5c (F = 0.9230, *p* = 0.4725) and I5d (F = 0.7060, *p* = 0.5746), *p* value was calculated with post-hoc Tukey HSD test (α = 0.05)].

**Figure 5:**
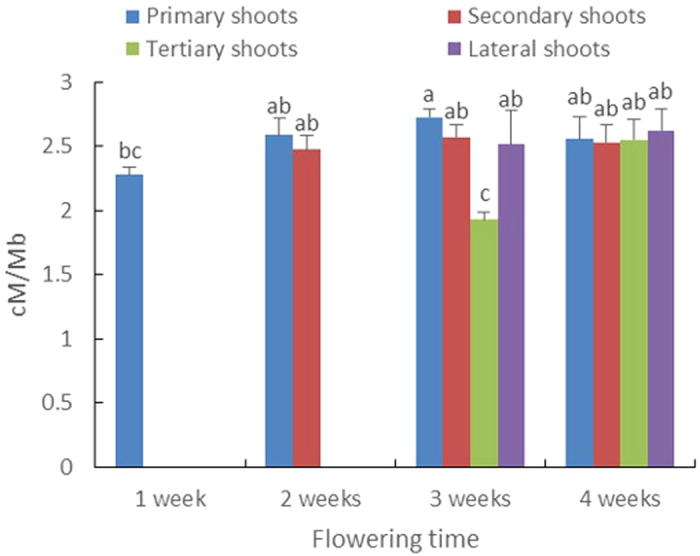
Mean recombination frequency of all nine intervals between four different shoot types at four time points during plant development. The mean recombination frequency calculated as cM_all_/Mb_all_. The individual of each interval is shown in Figure S2. Statistical significance was analyzed based on one-way ANOVA [F = 6.7630, *p* = 0.0001, *p* value was calculated with post-hoc Tukey HSD test (α = 0.05)].

In order to have an extensive overview on the spatio-temporal dynamics of male meiotic recombination frequency in all nine genomic intervals, we next compared the mean CO rate (cM_all_/Mb_all_) of all nine intervals between four different shoot types (primary shoot, secondary shoot, tertiary shoot and lateral shoot, Fig. 1) at four time points during plant development (Fig. 5). For both primary and tertiary shoots, the averaged CO frequency significantly increased over time [ANOVA: F = 6.7630, *p* = 0.0001, *p* value was calculated with post-hoc Tukey HSD test (α = 0.05)]. In the tertiary shoot, this CO increase amounted up to approximately 32% from the third to the fourth week upon flowering induction. In contrast, the averaged CO rate remained highly stable during plant aging in both secondary and lateral shoots, showing a constant rate around 2.5 cM/Mb. Strikingly, at the third week following flower induction, tertiary shoot show a significant lower recombination frequency compared to that of other three shoot types. Moreover, a similar decrease in CO rate was also observed in the primary shoot at the first week following flower induction. As such, these data reveal that both primary and tertiary shoots show similar dynamics in recombination frequency during shoot development, with young shoot displaying a generally lower recombination frequency that gradually increase during plant aging. In contrast, the overall male meiotic recombination frequency in secondary and lateral shoots remain highly stable during the whole period of flowering.

### Variation in age-dependent recombination rate dynamics in different genomic intervals is not correlated with their distance from the centromere

Our analyses reveal variation in the age-dependent CO frequency dynamics in the different genomic intervals tested, with some intervals showing an increase in CO rate during plant aging whereas other intervals displaying a rather constant CO rate over time. This variation suggests that a regulatory relationship between chromosome structure or genomic location and CO stability control in *Arabidopsis*. To explore this possibility, we next assessed the correlation between the meiotic recombination frequency change (ΔRF, ‘+’: increase/‘−’: decrease) of each shoot type for all intervals with the corresponding distance (Δ Distance) of their genomic location to the centromere (Table 2). Although in most shoot types there was a tendency for a positive relationship between the level of recombination frequency variation (ΔRF) and genomic distance of location to centromere (Δ Distance), the regression analysis revealed no correlation. Therefore, other regulatory factors may be involved in the determination of CO rate variability over time.

## Discussion

The pollen FTL system provides a fast and powerful tool to measure meiotic recombination frequency in *Arabidopsis* male sporogenesis^12^. As this system relies on the segregation analysis of hemizygous fluorescent markers in mature pollen grains, it is important that the fluorescent markers of each interval are correctly expressed. Considering their transgenic nature, we found that all FTL lines examined show a certain level of fluorescent loss in the corresponding DsRed, eYFP or eCFP fluorescent markers. Although the loss of fluorescent expression was generally low in most FTL intervals (around 1–4%), some intervals exhibited a much larger proportion of pollen that lost fluorescence. For example, the DsRed marker enclosed in the I1a interval displays a fluorescent loss rate of approximately 20%. This is potentially due to the location of the DsRed reporter near the centromeric region, putatively affecting the expression of transgenic reporter through epigenetic silencing.

Importantly, the inadvertent loss of fluorescence in each FTL interval may generate biases in the deduced recombination frequency, since this is calculated based on the quantification of pollen containing two or more fluorescent reporters^10^. The problem of fluorescence loss and associated biases in CO calculation can be bypassed by deploying the FTL system in the *quartet1-2 (qrt1-2*) mutant background, which allows to ignore tetrads containing non-fluorescent microspores, thereby effectively eliminating the erroneous segregation patterns from the analysis. By and large, we found that single-pollen analysis via flow cytometry is however most suitable for measuring recombination frequency for most FTL lines investigated, which allows high throughput fluorescent pollen analysis and generates much more data points to achieve statistically reliable analysis.

The frequency of meiotic recombination is strictly controlled at the molecular level^18^,^19^,^20^. In addition, recent studies have revealed that it is also influenced by both endogenous and external factors, including heterozygosity^21^,^22^,^23^, sex determination^7^,^17^,^24^ and ambient temperature^10^,^25^,^26^. Here, we show that the plant age and developmental position (shoot type) also influence the frequency of male meiotic recombination. More specifically, our results demonstrated that the recombination frequency significantly varies by plant age in four of the nine genomic intervals tested, with the other five intervals showing a similar trend of increased CO frequency during plant aging. Moreover, in line with the previous study using *Arabidopsis* FTL lines^10^, we found that interval I1a shows a significant increase in meiotic CO frequency in higher order branches. Strikingly, in six of the nine intervals tested, this positive correlation between branch order and male meiotic CO frequency was not observed, indicating that different genomic regions may show variable CO rate dynamics during shoot development. Furthermore, from the whole nine intervals view, both the primary and tertiary shoots show similar increasing dynamics of recombination frequency during shoot development. While in contrast, both secondary and lateral shoots remain highly stable during the whole period of flowering. All together, these findings suggest that there may exist inherent structural or regulatory differences (*in cis* or *in trans*) between different genomic regions that influence the dynamics of corresponding male meiotic recombination frequency during shoot branching and development. However, up till now, it is unclear which genomic or chromosomal features may underlie this region-specific variability in CO dynamics and stability stringency.

To determine genome-wide meiotic recombination frequencies in *Arabidopsis*, multiple regions in each of the five chromosomes need to be monitored. The FTL system, in this case, is an efficient method compared with other conventional methods, which are generally based on the segregation analysis of polymorphic molecular markers in a pool of individual progeny plants that result from a backcrossing of a hybrid to one of the corresponding parental lines. These genetic methods usually do not generate sufficiently large data sets to allow accurate determination of recombination frequency. Furthermore, thorough investigation of interactions between heterozygosity and meiotic recombination revealed that *Arabidopsis* F1 hybrids, resulting from the intercross between a Colombia-0 line and other 32 different accessions, show a significant variation in the male meiotic CO rate compared to their homozygote counterparts^27^. On the genomic level, it has been demonstrated that heterozygous regions show an increase meiotic CO rate when juxtaposed to homozygous regions^27^. All together, these findings demonstrate that genetic maps (i.e. representing the population-wide meiotic CO pattern) strongly depend on the genetic make-up and heterozygosity level of the plant(s) used for analysis. In contrast, the FTL system for male meiotic recombination measurement can be implemented in a fully homozygous background, and thus fully eliminates adverse effects of allelic diversity and heterozygosity.

In brief, our results provide new insights into the dynamics of male meiotic recombination frequency across different orders of inflorescence shoot branches. The results re-appraise the use of the FTL system, and provide a valuable data set that will be a benchmark for analyzing male meiotic recombination frequency in various genotypes or obtained under varying environmental conditions.

## Methods

### Plant material and growth conditions

*Arabidopsis thaliana* wild type accessions (Colombia-0 background) and *qrt1-2* (Colombia-3 background) were obtained from the Nottingham *Arabidopsis* Stock Center (NASC). All FTL lines were provided by G. P. Copenhaver upon request, all containing homozygous fluorescent markers in the *qrt1-2*^*−/−*^ background. For male meiotic recombination frequency analysis, hemizygous fluorescent marker lines were created by intercrossing homozygous FTL marker lines (either two or three fluorescent reporters linked on the same chromosome) with either Col-0 or *qrt1-2*^*−/−*^ diploid plants for either single pollen analysis or tetrad-based pollen analysis, respectively.

*Arabidopsis* seeds were vernalized at 4 °C for 2 days and germinated on K1 medium *in vitro* for 8 days at 20 °C under a 12 h/12 h (light/dark) photoperiod regime. Then, germinated seedlings were transferred to soil and cultivated in a controlled climate chamber under the same growth conditions (20 °C, 12 h/12 h light/dark). In order to stimulate flowering induction, photoperiod was changed into 16 h/8 h (light/dark) under the same conditions at the beginning of flower initiation.

Pollen isolated from flowers located at different shoot types were analyzed by microscopy or flow cytometry during plant development. For each shoot type, at least 200 tetrads were scored by epi-fluorescent microscopy or a minimum number of 2,000 single pollen grains was used for flow cytometry-based analysis. All analyses were performed in triplicate; i.e. each analysis included three biological replicates with pollen isolated from plants grown at the same time and under the same growth conditions.

### FTL genomic intervals

**Figure 6:**
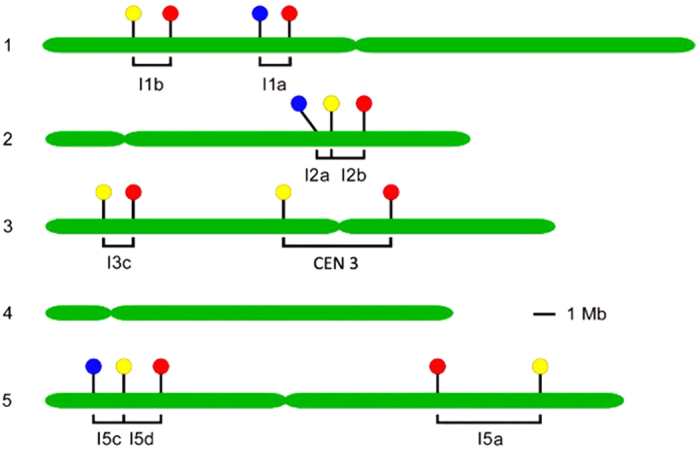
Map of fluorescent transgenes markers. The genomic location of the transgenic reporters encoding red (DsRed), yellow (eYFP), and cyan (eCFP) fluorescent proteins are indicated by filled circles on the five *Arabidopsis* chromosomes (green bars). The FTLs covering nine different genetic intervals (I1a, I1b, I2a, I2b, I3c, CEN 3, I5a, I5c and I5d) used in this study are delineated with brackets. The Arabidopsis Information Resource (TAIR) ‘chromosome map tool’ was used to scale the chromosomes and place the transgene insertion points on the physical map.

Nine independent FTL intervals (I1a, I1b, I2a, I2b, I3c, CEN3, I5a, I5c, and I5d) were used in this study to monitor male meiotic recombination frequency in *Arabidopsis thaliana*. These nine intervals span a series of genomic regions that range from 0.59 to 5.40 megabases (Mb) and that are randomly distributed along four of the five *Arabidopsis* chromosomes. All together, they cover 15.89% of the total genome (19.81 Mb), and they are located in either sub-telomeric, interstitial or centromeric regions (Fig. 6 and Table 3).

### Microscopy

Bright field and fluorescence microscopy and image capturing was performed using an Olympus IX81 inverted fluorescence microscope equipped with an X-Cite Series 120Q UV lamp and an XM10 camera (http://www.olympus.com/). Image processing and Z-stack projections were conducted using Olympus OlyVIA 2.4 software (http://www.olympus-sis.com).

### Flow cytometry

Single pollen analysis was performed using the BD FACSVerse flow cytometer equipped with a 488-nm and a 633-nm laser and eYFP and DsRed fluorescence was detected using 527/32-nm and 586/42-nm band-pass filters, respectively, based on the high-throughput fluorescent pollen analysis protocol of Yelina *et al*.^12^. The data were analyzed by BD FACSuite software and Microsoft Excel 2013.

### Recombination frequency (RF) quantification

Calculation of the Meiotic recombination frequency in each interval based on tetrad or single pollen grain analysis were performed by the following formulas:


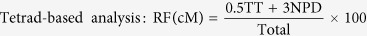


Where ‘TT’ is the number of tetratype, ‘NPD’ is the number of non-parental ditype and ‘Total’ is the number of all counted tetrads. The letter codes (a-l) for classification of tetrads used in this research are the same as those used in Copenhaver’s tetrad analysis, with FTL markers combined in a red–yellow–cyan configuration^11^.


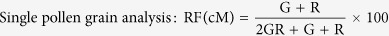


Where ‘G’ is the number of green-alone fluorescent pollen, ‘R’ is the number of red-alone fluorescent pollen and ‘GR’ is the number of pollen showing both green and red fluorescence.

### Data analysis

Data analysis and statistics were performed by Microsoft Excel 2013 and Data Processing System (DPS 7.05)^28^. One-way ANOVA with post-hoc Tukey HSD test was used for mean comparison of either two or multiple samples at the 5% significance level (α = 0.05).

## Additional Information

**How to cite this article:** Li, F. *et al*. Dynamics of male meiotic recombination frequency during plant development using Fluorescent Tagged Lines in *Arabidopsis thaliana. Sci. Rep.* **7**, 42535; doi: 10.1038/srep42535 (2017).

**Publisher's note:** Springer Nature remains neutral with regard to jurisdictional claims in published maps and institutional affiliations.

## Supplementary Material

Supplementary Figures

Supplementary Table S1

Supplementary Table S2

## Figures and Tables

**Table 1:** Fluorescent loss rate (%) of homozygous fluorescent transgene markers in different intervals under *quartet1-2* background.

Fluorescent markers	I1a	I1b	I2a	I2b	I3c	CEN 3	I5a	I5c	I5d
DsRed	19.05 ± 3.68	0.09 ± 0.17	/	3.77 ± 2.49	2.81 ± 1.87	3.75 ± 0.45	2.82 ± 2.13	/	0.99 ± 1.00
eYFP	/	0.87 ± 0.58	1.61 ± 1.40	1.61 ± 1.40	1.81 ± 1.34	1.80 ± 0.40	0.33 ± 0.34	0.16 ± 0.34	0.16 ± 0.34
eCFP	2.99 ± 1.05	/	1.79 ± 1.25	/	/	/	/	0.16 ± 0.34	/

The raw data of each intervals is shown in Table S1.

**Table 2:** Correlation between recombination frequency variation of shoots (ΔRF) with the map distance of interval to centromere (Δ Distance).

Intervals	Location (Mb)	Centromere location (Mb)	ΔDistance (Mb)	ΔRF (cM/Mb)			
				Primary	Secondary	Tertiary	Lateral
I1a	10.49	15.05	4.56	−1.70	−0.78	0.28	−0.18
I1b	4.83	15.05	10.22	0.86	−0.51	0.18	0.58
I2a	12.93	3.92	9.01	0.91	0.86	−1.86	0.73
I2b	13.95	3.92	10.03	1.20	1.03	0.22	0.22
I3c	3.72	13.80	10.08	1.77	1.03	0.31	0.81
CEN 3	13.81	13.80	0.02	0.34	−0.36	—	−0.15
I5a	20.62	11.88	8.74	0.31	−0.04	0.09	−0.05
I5c	3.07	11.88	8.81	1.06	1.09	0.30	0.12
I5d	4.63	11.88	7.25	0.63	−0.87	0.22	0.30

**Table 3:** The tested intervals of fluorescent transgenes markers.

Interval	Chr.	T-DNA 1	T-DNA 2	Mb	Location
I1a	1	9,850,022-eCFP	11,130,549-DsRed	1.28	Interstitial
I1b	1	3,905,441-eYFP	5,755,618-DsRed	1.85	Interstitial
I2a	2	12,640,092-eCFP	13,226,013-eYFP	0.59	Interstitial
I2b	2	13,226,013-eYFP	14,675,407-DsRed	1.45	Interstitial
I3c	3	3,126,994-eYFP	4,319,513-DsRed	1.19	Sub-telomeric
CEN 3	3	11,115,724-eYFP	16,520,560-DsRed	5.40	Centromeric
I5a	5	18,164,269-DsRed	23,080,567-eYFP	4.92	Interstitial
I5c	5	2,372,623-eCFP	3,760,756-eYFP	1.39	Interstitial
I5d	5	3,760,756-eYFP	5,497,513-DsRed	1.74	Interstitial
