# Supplementary material for: Dynamics of male meiotic recombination frequency during plant development using Fluorescent Tagged Lines in Arabidopsis thaliana
Source: Sci Rep. 2017 Feb 13;7:42535. doi: 10.1038/srep42535 (PMC5304153; doi:10.1038/srep42535)
Supplement: Supplementary Figures [file srep42535-s1.pdf]

Supplementary Figures

Dynamics of male meiotic recombination frequency during plant development using Fluorescent Tagged Lines in *Arabidopsis thaliana*

Fan Li, Nico De Storme, and Danny Geelen*

In vitro Biology and Horticulture, Department of Plant Production, Faculty of Bioscience Engineering, Ghent University, Ghent, 9000, Belgium
*corresponding. Danny.Geelen@UGent.be

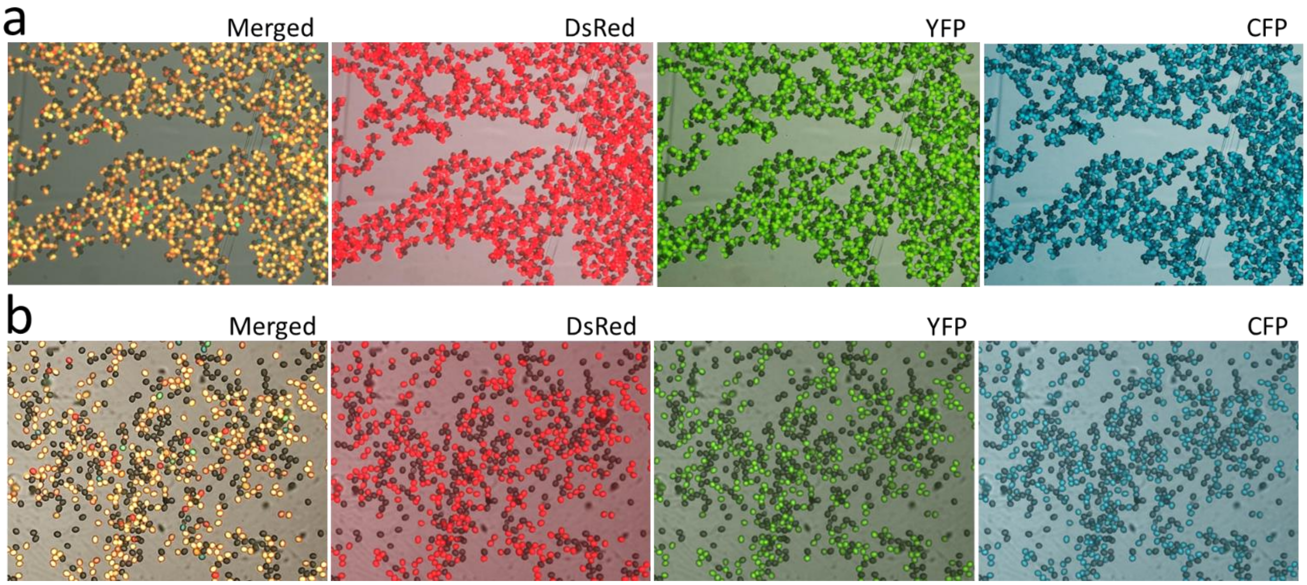

Figure S1. Micrographs of fluorescent pollen segregating for *I2ab* FTL T-DNAs. (a)Micrographs of *I2ab/*+++ *qrt1-2*$^{-/-}$ fluorescent tetrad pollens taken under DsRed, eYFP, and eCFP filters in bright field (BF), and a merged image. (b)Micrographs of *I2ab/*+++ single pollens taken under DsRed, eYFP, and eCFP filters in BF background. A merged image also shown.

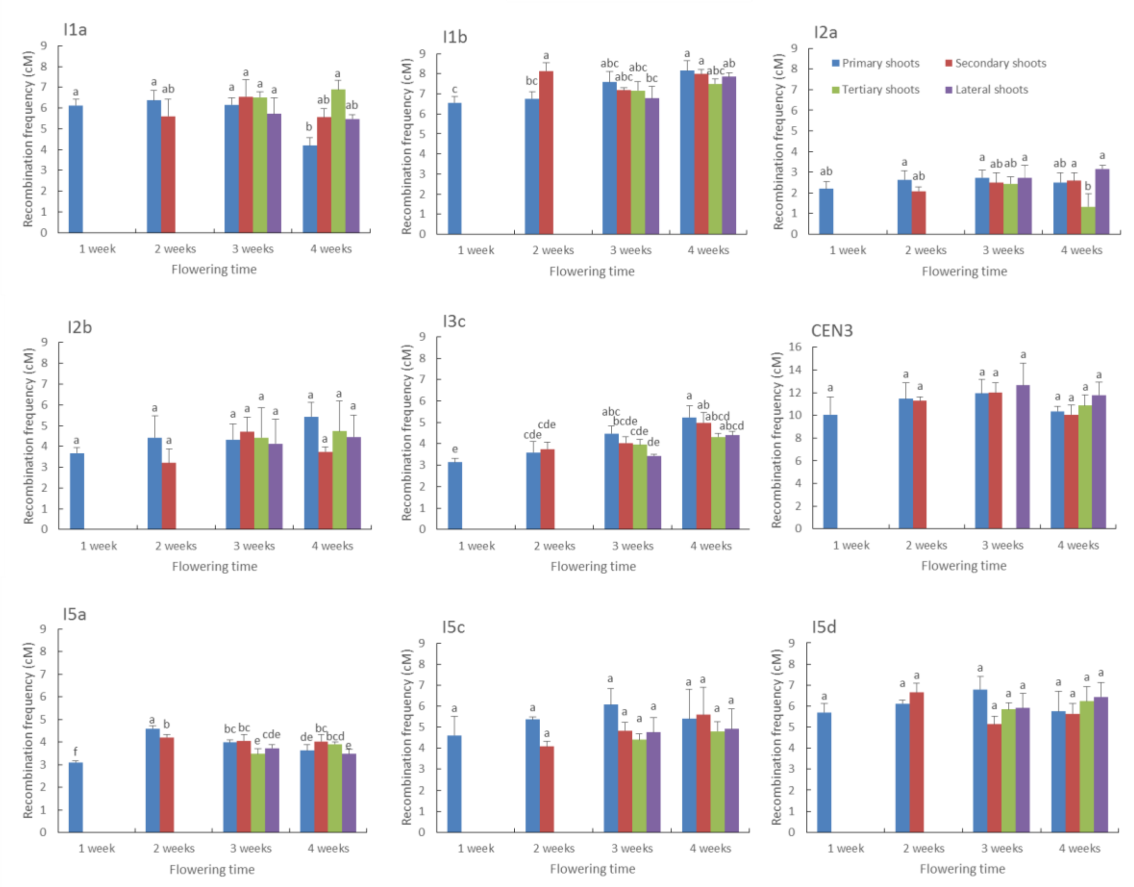

Figure S2. Male meiotic recombination frequency in different genomic intervals during the development of different shoot types in *Arabidopsis thaliana*. Statistical significance was analyzed based on one-way ANOVA [I1a (F=6.0840, p=0.0002), I1b (F=6.8380, p=0.0001), I2a (F=3.5920, p=0.0059), I2b (F=1.1990, p=0.3436), I3c (F=10.9220, p=0.0001), CEN3 (F=1.6920, p=0.1567), I5a (F=13.8560, p=0.0001), I5c (F=1.5650, p=0.1829) and I5d (F=2.1600, p=0.0636), p value was calculated with post-hoc Tukey HSD test (α=0.05)].
